# Finerenone: Questions and Answers—The Four Fundamental Arguments on the New-Born Promising Non-Steroidal Mineralocorticoid Receptor Antagonist

**DOI:** 10.3390/jcm12123992

**Published:** 2023-06-12

**Authors:** Luca Di Lullo, Carlo Lavalle, Alessia Scatena, Marco Valerio Mariani, Claudio Ronco, Antonio Bellasi

**Affiliations:** 1 Department of Nephrology and Dialysis, L. Parodi—Delfino Hospital, 00034 Colleferro, Italy; 2 Department of Clinical, Internal, Anesthesiologist and Cardiovascular Sciences, Sapienza University of Rome, Viale del Policlinico 155, 00161 Rome, Italy; carlo.lavalle@uniroma1.it (C.L.); marcoval.mariani@uniroma1.it (M.V.M.); 3 Diabetology Unit, San Donato Hospital, 52100 Arezzo, Italy; alessia.scatena@uslsudest.toscana.it; 4 International Renal Research Institute (IRRIV), S. Bortolo Hospital, 36100 Vicenza, Italy; 5 Department of Medicine, Division of Nephrology, Ente Ospedaliero Cantonale, 6900 Lugano, Switzerland; antonio.bellasi@eoc.ch

**Keywords:** type 2 diabetes mellitus, mineralocorticoid receptor antagonists, inflammation, fibrosis

## Abstract

Chronic kidney disease (CKD) is one of the most common complications of diabetes mellitus and an independent risk factor for cardiovascular disease. Despite guideline-directed therapy of CKD in patients with type 2 diabetes, the risk of renal failure and cardiovascular events still remains high, and diabetes remains the leading cause of end-stage kidney disease in affected patients. To date, current medications for CKD and type 2 diabetes mellitus have not reset residual risk in patients due to a high grade of inflammation and fibrosis contributing to kidney and heart disease. This question-and-answer-based review will discuss the pharmacological and clinical differences between finerenone and other mineralocorticoid receptor antagonists and then move on to the main evidence in the cardiovascular and renal fields, closing, finally, on the potential role of therapeutic combination with sodium-glucose cotransporter 2 inhibitors (SGLT2is).

## 1. Introduction

Despite current therapeutic options, patients with diabetic renal disease still have a residual risk of progression to end-stage renal disease with a related increased incidence of cardiovascular events. Beyond the metabolic and hemodynamic aspects, which are well controlled by currently available molecules (from inhibitors of the renin–angiotensin–aldosterone system, RAASi, to the co-receptor sodium/glucose transporter type 2 inhibitors, SGLT2i), are chronic inflammatory patterns. These are little explored but extremely important in determining renal and cardiovascular outcomes. Chronic inflammatory patterns present in patients can evolve into fibrosis, both renal and cardiac.

As of 2019, the American Diabetes Association (ADA) has recommended the use of SGLT2i in combination with angiotensin-converting enzyme inhibitors (ACEis) or angiotensin II receptor blockers (ARBs) with the purpose of slowing the progression of renal disease and reducing the impact of cardiovascular events in patients with diabetic nephropathy [1].

Since there is evidence of mineralocorticoid receptor (MR) hyperactivation in patients with chronic kidney disease and diabetes, which could lead to an increased rate of inflammation first and fibrosis later, the blockade of receptor activity could contribute to the lowering of residual cardiorenal risk in such a patient population [2].

The anti-proteinuric and anti-fibrotic effect of spironolactone, a steroid-type mineralocorticoid receptor antagonist (MRA), has long been known; however, such effectiveness has been burdened by a high incidence of side effects (gynecomastia and hyperkalemia above all) [2].

**Figure 1:**
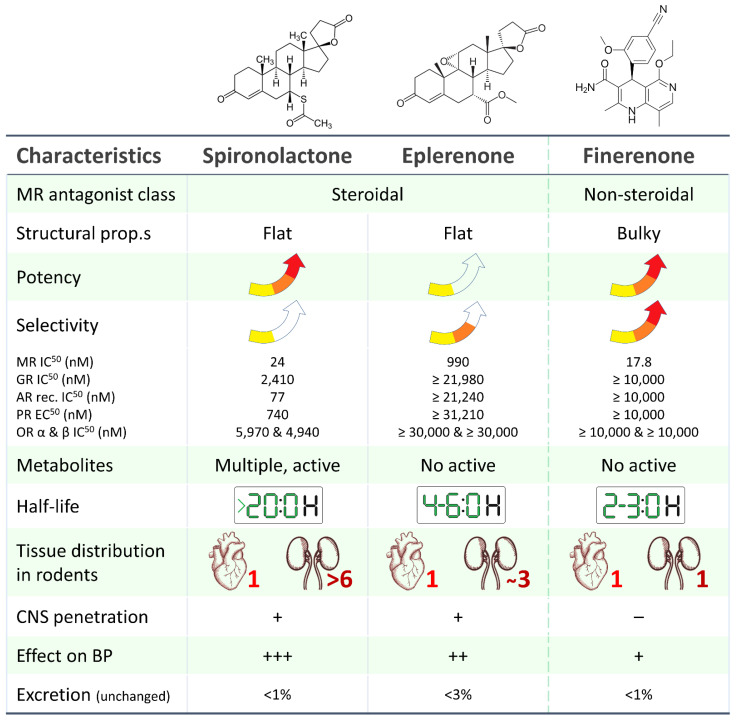
Main pharmacological and pharmacokinetic characteristics of steroidal (spironolactone, eplerenone) and non-steroidal (finerenone) mineralocorticoid receptor antagonists [4,5,6,7,8,9]. MR: mineralocorticoid receptor, AR: androgen receptor, GR: glucocorticoid receptor, PR: progesterone receptor, OR: oestrogen receptor.

Finerenone, in contrast to spironolactone and eplerenone, is a non-steroidal type MRA (hence the absence of gynecomastia as a side effect) with a high affinity (significantly higher than both spironolactone and eplerenone) for the mineralocorticoid receptor. It exhibits a balanced tissue distribution between the heart and kidney and, most importantly, is associated with a lower risk of hyperkalemia, as evidenced by the results of phase I and phase II clinical trial programs (Figure 1) [3].

## 2. Finerenone: Why Another MRA?

The mineralocorticoid receptor (MR) is a ligand-induced transcription factor expressed in many tissues, including the kidney, heart, and blood vessels. When aldosterone binds MR, it changes its conformation and translocates from the cytoplasm into the nucleus, binding specific hormone-response elements and recruiting transcriptional cofactors which act in the transcription or repression of target genes [10]. Nevertheless, MR is also responsible for a “non-genomic” activity modulating target molecules and pathways [11].

**Figure 2:**
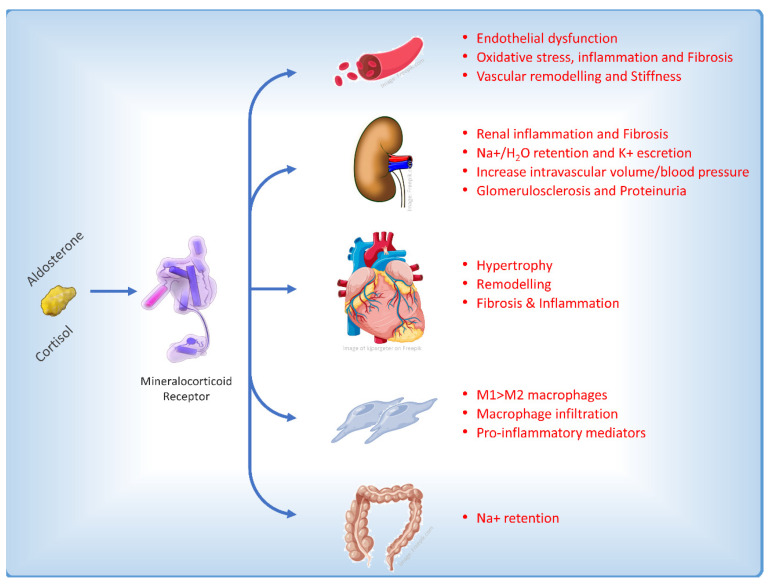
Main effects on different cellular types and pathophysiological pathways affecting the heart induced by the activation of the mineralocorticoid receptor [13].

The role of MR is well characterized in the distal nephron on aldosterone-sensitive kidney epithelial cells that express the 11b-hydroxysteroid dehydrogenase type 2 enzyme, which confers higher specificity of the MR for aldosterone versus cortisol. In these cells, MR activation is primarily involved in the regulation of blood pressure and sodium retention [2]. MR receptor is also expressed in kidney non-epithelial cells and in several other tissues including adipocytes, vascular smooth muscle cells (VSMCs), immune cells, and heart cells, in which it may be involved in regulation of cardiomyocyte growth and cardiac electrophysiology. In non-epithelial cells, MR is widely activated also by cortisol and its upregulation could stimulate fibrosis and inflammation, resulting in progression of renal and cardiovascular disease (Figure 2) [12]. 

In CKD experimental models, it was observed that aldosterone increased reactive oxygen species [14] and promoted mesangial apoptosis [15] and collagen synthesis [16]. This resulted in inflammation and cortical and medullary fibrosis. Injury of podocytes, a critical event in diabetic nephropathy, is prevented in mice by MR antagonism with eplerenone [17]. Autophagy, another essential process for podocyte maintenance and homeostasis, is also disrupted in CKD associated with type 2 diabetes (T2D) and spironolactone treatment increased podocytes autophagy in diabetic rats and restored podocytes autophagy under mechanical stress [18]. In endothelial cells, aldosterone promotes oxidative injury and endothelial disfunction [19,20] as MR activation is involved in vascular calcification and fibrosis, stiffness, and inflammation [21]. Furthermore, the activation of mineralocorticoid receptors (MR) has been found to have additional pathological effects on the immune system and pro-inflammatory cells. This includes the stimulation of the infiltration of pro-inflammatory M1 macrophages as opposed to M2 macrophages, increased proliferation of fibroblasts, the production of molecules that promote fibrosis [22], and a reduction in nitric oxide levels due to the influence of MR on the expression of the epithelial sodium channel in the endothelium [23].

In streptozotocin-induced diabetic rats and db/db mice, MR blockade with spironolactone, eplerenone, and finerenone reduces collagen deposition in glomerular, tubulointerstitial, and perivascular areas as well as decreased albuminuria [24,25,26].

In order to prevent severe comorbidities associated with T2D, such as nephropathy and end-stage renal disease (ESRD) and cardiovascular morbidity and mortality, preserving organ integrity and function is a challenge in patients with T2D [2].

The first steroidal MR antagonist (MRA), spironolactone, was developed as an antihypertensive drug with the intention to prevent sodium retention and decrease blood volume. However, because of its activities on progesterone and other nuclear receptors, it was observed that spironolactone causes relevant side effects, such as gynecomastia, impotence, and menstrual abnormalities. These side effects were at least partially ameliorated by the second-generation of steroidal MRA eplerenone [27]. Indeed, although it was observed in randomized control trials that steroidal MRAs (spironolactone and eplerenone) reduce the risk of hospitalization and cardiovascular death in patients with HF and reduced ejection fraction (HFrEF) [28,29], and received class IA recommendation by international clinical guidelines for the treatment of HFrEF [30], they are still underused, especially in patients with impaired kidney function [31], because of the risk of both acute kidney injury (AKI) and hyperkalemia. Furthermore, spironolactone is recommended by guidelines as an optimal fourth-line therapy in patients with resistant hypertension and estimated glomerular filtration rate (eGFR) >45 mL/min/1.73 m^2^ and serum K ≤ 4.5 mEq/L [32,33], but the risk of hyperkalemia represents the limit of use of MRAs also in this context [34,35]. 

As previously mentioned, MR activation concurs in the pathophysiology of CKD [36], and aldosterone is supposed to be one among main actors. Aldosterone levels were shown to start to increase when the glomerular filtration rate (GFR) is reduced by 50% [37] and another study showed that aldosterone levels are elevated up to fourfold in patients with a mean GFR of 27 mL/min per 1.73 m^2^ [38]. Additionally, during long-term blockade of the renin-angiotensin system (RAS), aldosterone breakthrough is seen [39]. The correlation between aldosterone levels, eGRF decline, and the aldosterone breakthrough phenomenon therefore supports aldosterone as a primary target for intervention in patients with CKD and T2D who are receiving a maximum-tolerated dose of ACEis or ARBs, shifting from RAS blockade to a renin-angiotensin-aldosteron system (RAAS) blockage. Several meta-analyses on steroidal MRAs in diabetic CKD reported an improvement in urinary albumin–creatinine ratio (UACR) but with a dramatic increased incidence of hyperkalemia [40,41]. Hou analyzed 16 studies of spironolactone added to routine antidiabetic, renoprotective, and antihypertensive treatment lasting 2–18 months and demonstrated a significant reduction of more than 60% in end-of-treatment 24-h urinary albumin/protein excretion, with the major concern of a more than a fivefold increased risk for hyperkalemia, particularly in those with impaired renal function [40]. Additionally, a 72-week intervention study with 4 arms combining irbesartan 150 or 300 mg daily with spironolactone 20 mg or placebo confirmed a long-term antiproteinuric effect (up to 30%) when combination was used [42]. More recently, prevention of CKD with spironolactone versus placebo was tested in the 3-year randomized PRIORITY study (Proteomic Prediction and Renin–Angiotensin–Aldosterone System Inhibition Prevention of Early Diabetic Nephropathy in Type 2 Diabetic Participants with normoalbuminuria). The study included 1777 patients with T2D and increased normal-to-mild albuminuria, of whom 218 had a high risk of CKD, as determined from a urinary proteomics-based risk pattern for CKD. To reduce the risk of hyperkalemia, only patients with a GFR > 45 mL/min/1.73 m^2^ were included. The urinary proteomic pattern predicted progression of both albuminuria and development of CKD stage 3b, and spironolactone was not able to prevent progression. Possible reasons for this result are the lack of statistical power, the short duration of the trial, or that the enrolled population was in too early a stage of disease for this mode of action to be effective [43]. 

Eplerenone, less potent but more selective than spironolactone, has documented benefits in HFrEF [29] and was considered a promising molecule for treating CKD in T2D patients because of the lack of hormonal side effects and antiproteinuric effects similar to those seen with spironolactone [44]. However, the increase in potassium levels resulted in a recommendation against eplerenone in T2D and CKD (US and UK labels).

Long-term studies with steroidal MRAs that aimed to prevent progression of established CKD have not been carried out, and therefore, whether the beneficial effect on albuminuria translates into prevention of ESKD is not known. 

Given the beneficial effect on proteinuria but considering hyperkalemia and the sexual side effects of steroidal MRAs, the efforts go towards the discovery and development of new non-steroidal MRAs (NS-MRAs) that are highly selective for MR, and at least equally effective than spironolactone and with a better safety profile. 

Finerenone is the first developed novel NS-MRA characterized by a unique binding mode (bulky) that determines high potency and selectivity for the MR, inhibits binding of both aldosterone and cortisol, and reduces recruitment of transcriptional cofactors in both the bound and not-bound conformational state of MR [2]. Indeed, the main difference among finerenone and spironolactone and eplerenone is the modulation of different transcriptional cofactors and expression/inhibition of a different genes profile. 

Differently from steroidal MRAs, finerenone has also tissue distribution (equal distribution between the heart and kidney), pharmacokinetic properties (a short half-life and no active metabolites), a greater MR selectivity than spironolactone, and a higher receptor binding affinity than eplerenone [6,45,46]. 

Preclinical studies provided preliminary evidence that finerenone modifies tissue remodeling by exerting anti-inflammatory, antifibrotic and antiproliferative actions on both the heart and the kidney [46,47]. 

The phase II clinical trials (ARTS, ARTS-DN, ARTS-HF) have shown a dose-dependent reduction in albuminuria with finerenone and a side-effects profile at least similar with the placebo or comparator, as well as smaller treatment-induced increases in serum potassium levels with finerenone compared with spironolactone [48,49,50]. ARTS (Miner Alocorticoid Receptor antagonist Tolerability Study) evaluated various doses of finerenone for 28 days in 393 patients with T2D, CKD, and chronic HF with a reduced ejection fraction. Compared with spironolactone, a comparable reduction occurred in N-terminal pro-brain natriuretic peptide and albuminuria, with a smaller increase in serum potassium, and a significantly lower rate of hyperkalemia (5.3% vs. 12.7%) and renal impairment (3.8% vs. 28.6%) was observed [48]. The ARTS-DN (ARTS in Diabetic Nephropathy) study evaluated the effect on albuminuria of different doses of finerenone and a placebo for 90 days in 823 patients with T2D and CKD on top of RAS inhibitor therapy. Treatment with finerenone led to a dose-dependent reduction in albuminuria (21–38%). Adverse events were comparable to those observed with the placebo. Hyperkalemia and subsequent discontinuation of the study drug occurred in 1.8% of patients receiving finerenone, compared with no patients on placebo. In addition, no differences in the incidence of an eGFR decrease of 30% were seen between the groups [49]. These data support the assumption that the antiproteinuric effect can be maintained with a limited effect on serum potassium. Finally, similar results were achieved in the ARTS-HF (ARTS in Heart Failure) study which evaluated finerenone versus eplerenone in subjects with T2DM, a worsening of chronic heart failure, and with or without CKD [50]. 

Furthermore, in large phase III trials involving patients with CKD and T2DM (Finerenone in Reducing Kidney Failure and Disease Progression in Diabetic Kidney Disease (FIDELIO-DKD) and finerenone in Reducing Kidney Failure and Disease Progression in Diabetic Kidney Disease (FIGARO-DKD)), finerenone significantly improved long-term kidney and cardiovascular outcomes and albuminuria as compared with the placebo, on top of RAS inhibition [51,52]. Although hyperkalemia was increased, values that required permanent discontinuation of finerenone were very low (2.3% for FIDELIO-DKD and 1.2% for FIGARO-DKD) These companion studies have been the first to test if the antiproteinuric effect of aldosterone blockade translates safely into prevention of progression of kidney disease and prevention of CV events. 

CKD and diabetes independently increase the risk of atrial fibrillation (AF), as well as the risk of adverse CV outcomes in patients with AF; therefore, it is intriguing that in the FIDELIO-DKD study, new-onset AF occurred in 82 patients (3.2%) treated with finerenone versus 117 patients (4.5%) receiving the placebo (HR = 0.71; 95% CI = 0.53–0.94; P = ¼ 0.016) [53].

Finally, FIDELITY (finerenone in Chronic Kidney Disease and Type 2 Diabetes and combined FIDELIO-DKD and FIGARO-DKD trial program analysis) is the prespecified pooled-analysis of both phase III trials that included 13,026 patients with a mean follow-up of 3 years. Both composite cardiovascular (CV death, non-fatal myocardial infarction, non-fatal stroke, or hospitalization for heart failure) and kidney (kidney failure, sustained > or =57% decrease in eGFR from baseline over > or =4 weeks, or renal death) outcomes were significantly reduced by finerenone (14% and 23%, respectively) with a really low incidence of permanent drug discontinuation due to hyperkalemia events (1.7%) [2]. In summary, all reported evidence underlines both the clinical need of MR inhibition in the treatment of CKD in T2DM and the possibility that new NS-MRAs represent an effective tool with a better handling and risk/benefit ratio.

## 3. Finerenone and Cardiovascular Outcome: Only Diuretic or Something Else?

The activation of renin–angiotensin–aldosterone system (RAAS) plays a key role in the pathophysiology of cardiorenal syndrome, leading not only to salt-water overload and systemic congestion, but also to structural remodeling of cardiovascular (CV) system. Mainly activated by aldosterone, the mineralocorticoid receptor (MR) has major effects on fluid and electrolyte regulation, blood pressure, inflammation, and fibrosis. It is implicated as main effector of the detrimental consequences of neuro-hormonal activation seen in several CV and renal conditions [54]. 

**Figure 3:**
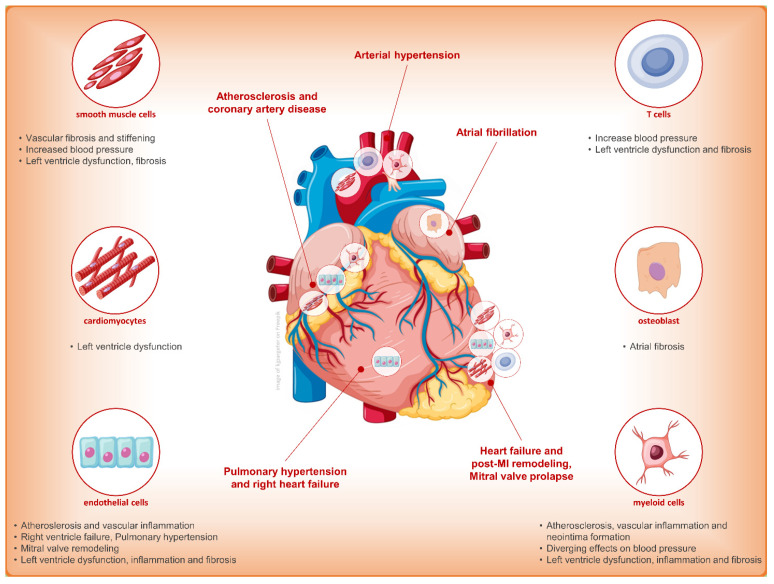
Main effects on different cellular types and pathophysiological pathways affecting heart induced by the activation of mineralocorticoid receptor [55].

As shown in Figure 3, the heart is particularly full of MRs that are associated with altered extracellular matrix regulation, increased M1 (pro-inflammatory) macrophage phenotype, release of pro-fibrotic mediators related to collagen synthesis and oxidative stress, and release of pro-inflammatory cytokines and mediators [55]. In particular, aldosterone demonstrated negative effect on cardiac hypertrophy and fibrosis in mouse models of heart failure and post-myocardial infarction remodeling. MR deletion resulted in smaller scar volume, less myocardial fibrosis, and LV function improvement. These detrimental effects of aldosterone are mediated by enhanced oxidative stress and myocyte apoptosis due to MR activation on myeloid cells, leading to a shift towards the more-destructive M1 macrophage response, and to MR activation on smooth muscle cells (SMCs) resulting in proliferation and fibrosis [56]. In models of chronic HF, aldosterone resulted associated with negative cardiac remodeling, pressure overload-induced hypertrophy, inflammation, and fibrosis via MR activation on myeloid cells, T cells, and endothelial cells [57,58]. The MR-mediated cardiac remodeling is mainly produced by inflammatory and fibrotic effector molecules galectin 3 and lipocalin 2 [59]. MR activation plays a role in the pathophysiology of HF with preserved EF (HFpEF) by increasing oxidative stress and inflammation, which hampers nitric oxide (NO) production, thus resulting in perivascular fibrosis and myocardial relaxation impairment. With similar pro-inflammatory effects, a reduction in NO availability and monocyte recruitment at endothelial level, MR activation favors atherosclerosis development [60]. Moreover, the impairment of endothelium-dependent vascular relaxation and the upregulation of L-type Ca channels on SMCs are responsible for the impact of aldosterone on arterial hypertension and on vascular stiffness [61]. Lastly, the imbalance of endothelin-1 signaling pathway and paracrine crosstalk in endothelial cells produced by MR activation may play a role in the development of pulmonary hypertension [62]. 

In a subset of patients treated with angiotensin-converting enzyme inhibitors and/or angiotensin-receptor blockers, serum aldosterone levels are increased, due to a phenomenon called “aldosterone breakthrough”, associated with the above-mentioned detrimental effects on CV system [39]. In consideration of MR-induced CV inflammation, fibrosis, and remodeling, steroidal mineralocorticoid receptor antagonists (MRAs), as such spironolactone and eplerenone, gained a central role in the treatment of CV conditions associated with RAAS upregulation, as heart failure with reduced ejection fraction (HFrEF) and resistant hypertension. The RALES trial showed that spironolactone use was related to a 30% all-cause mortality reduction in HFrEF and New York Heart Association (NYHA) class III-IV symptoms [28]. In the PATHWAT-2 trial, treatment with spironolactone was superior to treatment with bisoprolol, doxazosin, or a placebo for blood pressure reduction on top of three, maximally tolerated medication in resistant hypertension [63]. However, spironolactone treatment was not associated with significant reduction in CV death, cardiac arrest, or HF hospitalization in patients with HFpEF (FE ≥ 45%) in the TOPCAT trial [64].

In the small, randomized AREA-IN-CHF trial, which included patients with heart failure with reduced ejection fraction (HFrEF), canrenone, a metabolite of spironolactone, demonstrated a reduction in both mortality rate and remodeling [65]. The second generation, a steroid-based MRA eplerenone reduced mortality and morbidity on top of optimal medical therapy in patients with post-MI HFrEF [29]. At the same time, in the EMPHASIS-HF trial, adding eplerenone on top of medical therapy resulted in a statistically significant reduction in the risk of death and hospitalization (HR 0.63, 95% CI 0.54–0.74) in patients with chronic HFrEF [66]. Relative to spironolactone, eplerenone treatment was associated with lower rate of hormonal side effects, probably due to the much higher specificity for the MR leading to reduced cross-reactivity with antagonism at the androgen receptor [66].

On the basis of these landmark trials, current HF guidelines recommend the use of MRAs in patients with HFrEF [67]. However, steroidal MRAs remain under-prescribed in daily clinical practice due to the perceived risk of hyperkalemia, worsening of glomerular filtration rate, as well as hormonal side effects including erectile dysfunction, menstrual irregularities, and gynecomastia [68,69]. To avoid these adverse effects, developing MRAs from a non-steroidal base compound has gained much interest. These compounds have high affinity, improved MR specificity, and improved therapeutic index, theoretically resulting in a reduced risk of hyperkalemia and unwanted off-target effects. Finerenone is a third-generation, dihydropyridine derivative, non-steroidal MRA which acts as a bulky, inverse agonist, decreasing MR nuclear translocations as well as co-factor and RNA polymerase recruitment compared to steroidal MRAs [13]. Preclinical studies showed the beneficial effects of finerenone on cardiovascular system, possibly mediated by inhibition of pro-fibrotic and pro-inflammatory genes. Finerenone was found to inhibit basal expression of MR-target gene Sgk1 that has been associated with the development of hypertension, fibrosis, diabetes, and metabolic syndrome [70]. In a mouse model of CKD induced by nephrectomy with consequent cardiac failure, finerenone improved cardiac function and decreased fibrosis [46]. Moreover, by inhibiting aldosterone-induced upregulation of connective growth factor and of the lysyl oxidase, finerenone prevented left ventricular dilatation and cardiac dysfunction development. Decreasing plasma levels of matrix metalloproteinase-2, level of superoxide anions and aldosterone-induced apoptosis and SMCs proliferation rates, finerenone was found to decrease arterial stiffness and neointimal hyperplasia [71]. As compared to eplerenone, finerenone reduced cardiac fibrosis and improved cardiac strain patterns in a mouse model of isoproterenol-induced cardiac fibrosis. Finerenone use was associated with lower concentration of macrophages and profibrotic cytokines probably due to the reduced expression of profibrotic tenascin gene in the myocardial tissue [46]. Lastly, finerenone-treated mice showed a reduced expression of brain natriuretic peptide (BNP) and cardiac troponin T in a model of pressure overload as compared to eplerenone-treated mice [72]. The differences in cardioprotective effect and safety of finerenone as compared to steroidal MRAs may be related to the intrinsic mechanism of action. Finerenone acts as an inverse agonist, inhibiting co-regulator recruitment even in the absence of aldosterone, whereas steroidal MRAs serve as partial agonists, resulting in some level of co-regulator recruitment at high concentrations [13]. Moreover, the MRs in the heart are concentrated in non-epithelial cells, such as myeloid cells and macrophages, and can be activated by both aldosterone and cortisol leading to pathological remodeling. However, in contrast to steroidal MRAs, which are more concentrated in the kidneys, finerenone is equally distributed between the heart and kidneys, explaining its superiority over steroidal MRAs in cardioprotection, with less incidence of hyperkalemia derived by MR blockage in renal epithelial cells [73]. Pitt et al. demonstrated that finerenone was associated with a lower incidence of hyperkalemia as compared to spironolactone in patients with HFrEF [48], whereas the ARTS-HF showed that the incidence of death of any cause and adverse cardiac events (including hospitalizations and emergency presentation for HF) were lower in finerenone arms than in eplerenone after 90 days follow-up, in patients presenting with worsening HFrEF and either CKD, T2DM, or both [50].

In 2021, FDA approved finerenone for use in patients with CKD in the setting of type 2 diabetes mellitus (T2DM) on the basis of two landmark phase-III trials, the FIDELIO-DKD trial and the FIGARO-DKD. In the FIDELIO-DKD trial [34,52], finerenone reduced the composite outcome of death from cardiovascular causes, nonfatal myocardial infarction, nonfatal stroke, or hospitalization for heart failure as compared to the placebo (hazard ratio, 0.86; 95% CI, 0.75 to 0.99; *p* = 0.03). On the same line, the FIGARO-DKD trial [52] showed the superiority of finerenone in reducing the risk of death from cardiovascular causes, nonfatal myocardial infarction, nonfatal stroke, or hospitalization for heart failure as compared to the placebo (hazard ratio, 0.87; 95% confidence interval [CI], 0.76 to 0.98; *p* = 0.03). The prespecified FIDELITY analysis [74] combining the FIDELIO-DKD and FIGARO-DKD trials confirmed a statistically significant reduction in a composite cardiovascular outcome associated with finerenone use (HR 0.86, 95% CI 0.78–0.95), mainly driven by reductions in HF hospitalizations (HR 0.78, 95% CI 0.66–9.92). These studies underline the cardioprotective effect of finerenone in CKD patients with T2DM, a population at high risk of CV disease development, and are thought provoking with regards to the possible role of finerenone in the prevention of the development in symptomatic HF. Although patients with symptomatic HFrEF were not included in the landmark trials, almost 8% of patients in FIDELITY had a history of HF and a subgroup analysis of FIDELIO-DKD trial showed that finerenone reduced the composite cardiovascular outcome irrespective of baseline HF history [75]. Moreover, finerenone was shown to reduce the risk of new-onset HF (HR 0.86, 95% CI 0.50–0.93) and a new-onset atrial fibrillation or flutter (HR 0.71, 95% CI 0.53–0.94) in sub-analysis of the landmark trials [53,76]. These sub-analyses suggest that finerenone may blunt the pro-inflammatory and pro-fibrotic effects of MR, preventing and slowing the progression of atrial and ventricular detrimental remodeling associated with aldosterone and eventually related to the development of HF and AF. Interestingly, 6.7% and 7.2% of patients in FIDELIO-DKD trial were taking SGLT2 inhibitors and glucagon-like peptide-1 (GLP-1) receptor antagonists, but the small sample size does not allow solid conclusions on the cardiovascular effects of an association therapy of these drugs with finerenone to be drawn [77,78]. Although finerenone was associated with beneficial effects on cardiovascular system in patients with CKD and T2DM, future research should address its use in patients with HFrEF (formally not included in the landmark trials) and in patients with HFpEF. The ongoing study to evaluate the efficacy and safety of finerenone on morbidity and mortality in participants with heart failure and left ventricular ejection fraction greater or equal to 40% (FINEARTS-HF) is currently investigating the therapeutic benefit of finerenone versus a placebo in HFpEF patients with cardiovascular death and heart failure events as the primary outcome (trial NCT identifier: NCT04435626; https://clinicaltrials.gov/ct2/show/NCT04435626 accessed on 28 April 2023). In conclusion, finerenone proved favorable results in terms of CV protection and ongoing trials will clarify its potential role as a new weapon against CV disease. 

## 4. Finerenone and Diabetic Nephropathy: A New and Ultimate Cornerstone?

Diabetic disease is the leading comorbid factor in incident patients undergoing chronic dialysis treatment, and renal involvement still develops in 40% of diabetic patients with a consequent increase in the incidence of cardiovascular complications [79].

Over the past 20 to 30 years, nephrologists have tried to apply therapeutic strategies to achieve the dual purpose of slowing progression of renal damage and preventing cardiovascular comorbidities [80,81].

The advent of drugs able to antagonize the renin–angiotensin–aldosterone system (RAASi) undoubtedly represented an important first step forward in the attempt to delay the progression of renal disease, but it was not enough. Both ACE-converting enzyme blockers (ACE-inhibitors, ACEi) and angiotensin II receptor antagonists (ARBs) have certainly represented a milestone in the management of diabetic patients with hypertension and proteinuria of varying degrees as well as heart failure patients [80,81].

What, however, RAASi have not been able to completely stop is the so-called “residual risk” of progression to end-stage renal disease, in addition to the risk of developing hyperkalemia and the potential risk of worsening renal function when used improperly (ESRD) [80,81,82]. Moreover, in up to 40% of patients and after several weeks of ACEi or ARBs treatment, plasma aldosterone returns to pre-treatment levels, carrying on all its negative effects in terms of inflammation and fibrosis. This phenomenon is called “aldosterone brealthrough” [39].

The recent advent of new classes of oral hypoglycemic drugs, such as sodium/glucose co-transporter type 2 (SGLT2i) inhibitors and glucagon-like peptide type 1 (GLP-1) agonists, has brought a first breath of fresh air after years of “pharmacological paralysis” by demonstrating that these molecules are capable of slowing the progression of renal damage in the diabetic patient and reducing the incidence of cardiovascular events [83].

In addition, residual renal risk is also associated with the risk of death from cardiovascular causes, even with an optimal pharmacological treatment with mineralocorticoid receptor antagonists (MRAs). However, aside from providing a reduction in proteinuria, this comes at the expensive of a risk of hyperkalemia [84,85]. 

Finerenone, a new MRA, is a candidate for playing a primary role in the field of nephroprotection of the diabetic patient partly due to its pharmacological properties.

Even before the program of phase-3 studies with finerenone (FIDELIO-DKD, FIGARO, and pooled-analysis FIDELITY) aimed at evaluating the renal and cardiovascular effects of finerenone treatment, several data have been published regarding the nephroprotective action of MRAs. These findings have particularly focused on the effects of spironolactone, eplerenone, and finerenone on urinary albumin excretion (UACR). These experiences showed that MRAs had a greater impact than other RAASi in reducing UACR levels in both diabetic and non-diabetic patients [43,84]. FIDELIO-DKD is a randomized, double-blind, versus placebo study aimed at evaluating the efficacy and safety profile of finerenone on renal and cardiovascular outcomes in a population of patients with diabetic nephropathy [34].

The study enrolled 5734 patients with type 2 diabetes mellitus (T2DM) treated at the highest dosage of ACEi or ARB with UACR values between 300 and 5000 mg/24 h and eGFR between 25 and 75 mL/min/1.73 m^2^ or patients with UACR between 30 and 300 mg/24 h, an eGFR between 25 and 60 mL/min/1.73 m^2^, and a diagnosis of diabetic retinopathy [34,86]. The composite primary outcome was determined by renal failure (defined as terminal kidney disease, i.e., eGFR ≤ 15 mL/min), sustained reduction of eGFR by at least 40% from baseline over a period of time greater than or equal to 4 weeks, and death from renal causes [34,86]. In the follow-up period, an event referable to the primary outcome described above was documented in 17.8% of patients treated with finerenone and 21.1% of patients in the placebo group [34,86] and a sustained reduction of UACR level was also achieved. Patients who received finerenone had a higher mean serum potassium level than those who received placebo: the maximal difference of 0.23 mmol per liter was observed at month 4, and the difference remained largely stable thereafter.

Hyperkalemia-related adverse events were overall more frequently with finerenone than with placebo (18.3% and 9.0%), but the frequency of hyperkalemia leading to discontinuation of the investigational drug was low in both groups (2.3% and 0.9%, respectively) and no fatal hyperkalemia adverse events were reported.

The FIGARO-DKD study, in contrast to FIDELIO but with the same design, started from a different background and had as its primary endpoint a cardiovascular composite (interval of time to death from cardiovascular causes, non-fatal myocardial infarction, nonfatal stroke or hospitalization for heart failure) [34,86,87]. FIGARO-DKD enrolled 7437 patients with T2D and CKD, defined as those with an UACR of 30–300 mg/g and an eGFR 25–90 mL/min/1.73 m^2^ (CKD stage 2–4) or an UACR of ≥300 mg/g and an eGFR ≥ 60 mL/min/1.73 m^2^, including therefore more patients with earlier-stage CKD and T2D than in the FIDELIO-DKD. During a median follow-up of 3.4 years, a primary outcome event occurred 12.4% of patients treated with finerenone and 14.2% in the placebo. The incidence of hyperkalemia was higher in the finerenone-treated arm (10.8%) than in the placebo-treated arm (5.3), but treatment discontinuation rate was also low in both arms [76].

Considering the complementary populations of FIDELIO-DKD and FIGARO-DKD, these findings demonstrate that finerenone has a cardiorenal protective effect across a large population of T2D patients with CKD. FIDELITY was a compendium of data from FIDELIO and FIGARO with the purpose of testing the efficacy and safety of finerenone in patients with T2DM, stage 1 to 4 CKD, and moderate-to-severe albuminuria. The pooled analysis included more than 13,000 patients and finerenone was shown to reduce the composite renal endpoint by 23%, a finding that was also confirmed in the analysis of different patient subgroups. In addition, finerenone was found to reduce the risk of developing ESRD that required the initiation of dialysis therapy by 20% [74]. FIDELITY data also documented a 14% reduction in the cardiovascular composite endpoint and a 22% reduction in the risk of hospitalization for heart failure.

**Figure 4:**
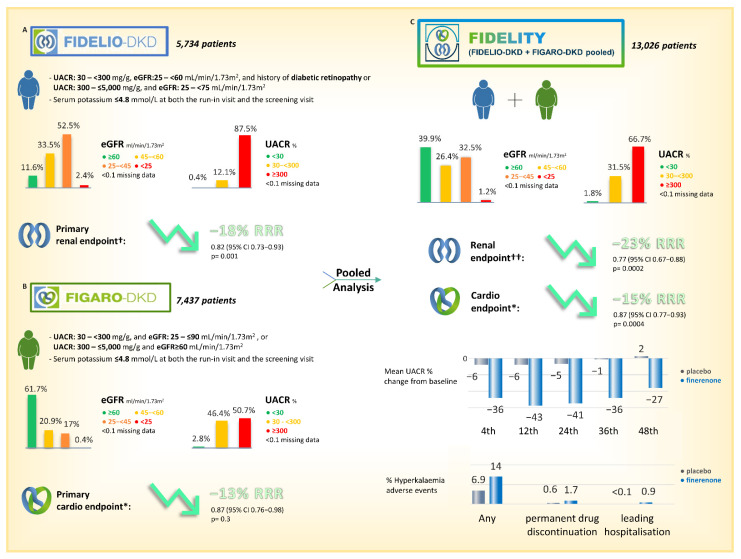
Finerenone phase III trials and pooled analysis at glance. (**A**): Finerenone in Reducing Kidney Failure and Disease Progression in Diabetic Kidney Disease (FIDELIO-DKD); (**B**): Finerenone in Reducing Cardiovascular Mortality and Morbidity in Diabetic Kidney Disease (FIGARO-DKD); (**C**): Combined FIDELIO-DKD and FIGARO-DKD Trial program analysis (FIDELITY). † Composite renal outcome: kidney failure (ebd-stage kidney disease or sustained decrease in eGFR to <15 mL/min/1.73 m^2^), sustained ≥40% decrease in eGFR from baseline, or renal death. * Composite cardio outcome: death from cardiovascular causes, non-fatal myocardial infarction, non-fatal stroke, hospitalization for heart failure. †† Composite renal outcome: kidney failure (ebd-stage kidney disease or sustained decrease in eGFR to <15 mL/min/1.73 m^2^), sustained ≥57% decrease in eGFR from baseline, or renal death.

In FIDELITY, the occurrence of hyperkalemia was higher (14%) in the finerenone-treated group than in the placebo group (6.9%), but according to the previous mentioned studies, the rate of treatment discontinuation due to hyperkalemia dropped to 1.7% and 0.6%, respectively, and to 2.4% (versus 0.8% in the placebo group) and 0.6% (versus 0.3% in the placebo group) in patients with eGFR < 60 mL/min/1.73 m^2^ or > 60 mL/min/1.73 m^2^, respectively (Figure 4) [74].

In conclusion, it can be said that the use of finerenone in patients with proteinuric diabetic nephropathy in therapy with the maximum recommended dosage of RAASi is accompanied by a slight increase in serum potassium levels and low incidence of unmanageable hyperkalemia leading to treatment interruption [88].

Of course, we will wait for “real world” data that should tell us how finerenone therapy will impact the quality of life of patients with diabetic kidney disease and, most importantly, renal disease progression together with cardiovascular outcomes.

## 5. Finerenone and Perspectives: Stand Alone or in SGLT2i Joint Future for Renoprotection?

The availability of new products such as SGLT2i, GLP-1RA, non-steroidal MRA finerenone, and potassium binders suggests a new era in the treatment of diabetic nephropathy as well as new patients’ perspectives. In this scenario, the main question is whether the combination of these new therapies provides additional benefits beyond what was demonstrated in the registration trials of each compound. In particular, should the new non-steroidal MRA finerenone and SGLT2i be used alone or in combination? Unfortunately, no study has investigated this question and no conclusive answer can be given. However, some considerations can be made based on available experimental data. 

Although the mechanism(s) is still unclear, SGLT2i renoprotection may be due to a change in renal hemodynamic. The inhibition of tubular glucose and sodium reabsorption by SGLT2 inhibitors (SGLT2i) has the potential to restore the impaired tubuloglomerular feedback observed in individuals with diabetes and chronic kidney disease. This restoration can lead to a reduction in glomerular hypertension and hyperfiltration [89,90]. Although these hemodynamic changes cause an initial eGFR reduction (eGFR dip), the long-term effect is a slower rate of eGFR decline in SGLT2i users [91,92]. Aside of the impact on glomerular hemodynamics, the inhibition of glucose and sodium reabsorption in proximal tubule may attenuate the metabolic stress of kidney cells [93] and mimic a state of medullary hypoxia, leading to an increase in the generation of hypoxia protective factors and improving cortical oxygenation [94,95]. Furthermore, a metabolic shift towards increased lipolysis and ketogenesis as well as anti-inflammatory and antioxidative properties has been described and postulated as a potential mechanism to explain the renoprotective effects of SGLT2i [96,97]. 

As discussed in the previous sections, finerenone exerts its renoprotective activity through both hemodynamic and non-hemodynamic pathways. The rapid effect on eGFR and albuminuria can be seen when the treatment is started; moreover, the early separation of curves of a cardiovascular endpoint may suggest an hemodynamic effect of finerenone [98]. Mineral receptors (MR) are expressed by several cell types of both epithelial and non-epithelial origin [6,36,99]. MR antagonism on non-epithelial cells (including fibroblasts, vascular smooth muscle cells, podocytes, and inflammatory cells) explain the anti-inflammatory and anti-fibrotic activities of finerenone in the kidneys and across multiple organs level [100,101].

**Figure 5:**
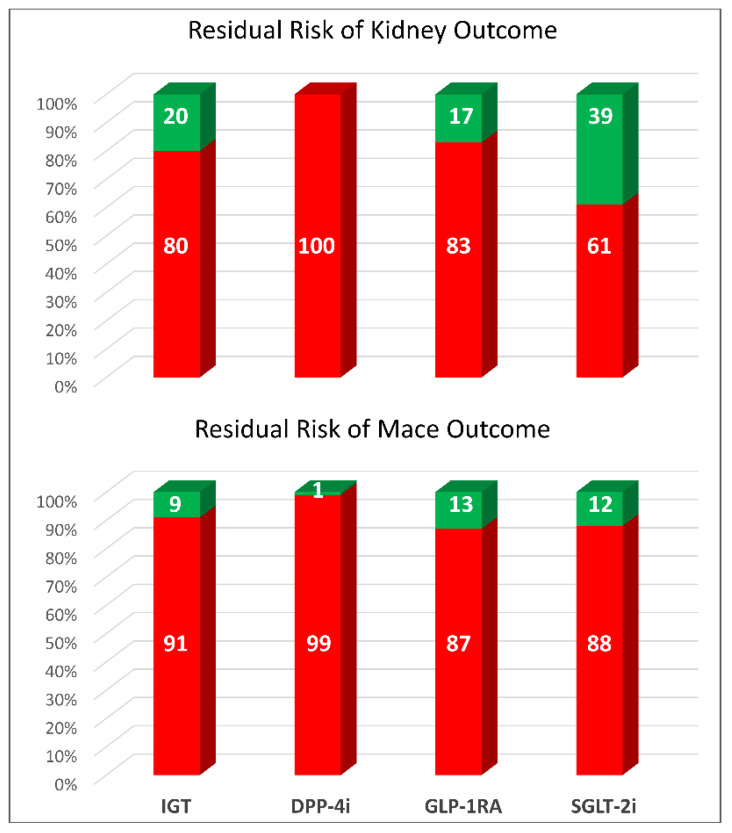
Residual and removed risks of kidney outcomes (generally, sustained decline in eGFR at least 50%, ESKD, and death from renal causes) and major adverse cardiovascular events (MACE) in intensive glucose trials (IGT) and in cardiovascular outcome trials with newer drugs. Dipeptidyl-peptidase-4 inhibitors (DPP-4i), glucagon-like peptide-1 receptor agonist (GLP-1RA), and sodium-glucose transporter-2 inhibitors (SGLT-2i) [102].

Some lines of evidence support the notion that combination therapy of SGLT2i and finerenone may have a synergic effect through common and distinct pathophysiological pathways and possibly will reduce the residual cardio–renal risk noted after the registration trials of main molecules (Figure 5) [102].

Preclinical data showed that in a model of hypertension-induced, end-organ damage (hypertensive, N(ω)-nitro-L-arginine methyl ester-treated, renin-transgenic (mRen2)27 rats), the combination of a low-dose of finerenone (1 mg/kg) and empagliflozin (3 mg/kg) was superior to both a low-dose or a standard-dose of each monotherapy. With an about 50% survival rate in placebo-treated rats over the course of 7 weeks, the combination therapy provided a 93% survival rate compared to a 60/70% survival rate of empagliflozin 3 or 10 mg/kg alone and about an 85% of finerenone 1 or 3 mg/kg alone. [103]. Furthermore, proteinuria was also reduced by 86% among rats treated with the combination therapy, whereas it was reduced by 27% and 38% in the low-dose finerenone and empagliflozin monotherapies study arms, respectively. Notably, the use of standard-dose monotherapies did not prove to be superior in proteinuria reduction to combination therapy (−87% and −64% proteinuria reduction for finerenone and empagliflozin, respectively) [103]. Similarly, although the administration of each monotherapy provoked a dose-dependent protection of cardiac and kidney fibrosis, low-dose combination therapy was again more efficacious than the respective monotherapy [103]. This synergic effect is also supported by the exploratory ROTATE-3 trial which combines dapagliflozin treatment with eplerenone in an open-label, cross-over trial on 46 patients with CKD. The combination therapy resulted in a significant addictive urinary-to-albumin-creatinine ratio (UACR) decrease, as well as a significant reduction in the occurrence of hyperkaliemia events when compared to eplerenone monotherapy (4.3% for dapagliflozin + eplerenone vs. 17.4% for eplerenone alone) [104]. The analysis of a patient using a sodium-glucose cotransporter 2 inhibitor (SGLT2i) in the combined FIDELIO-DKD and FIGARO-DKD Trial program, known as FIDELI-TY, demonstrates promising and reassuring data [105]. About 6.7% of 13,026 patients included in FIDELITY were also treated with an SGLT2i at baseline and an additional 8.5% of the recruited subjects also initiated an SGLT2i during the trial. Finerenone reduced the primary cardiovascular endpoint as well as the secondary key renal outcome, independently of the use of SGLT2i at baseline or during the trial. Although a small number of patients did not allow to test whether the combination of SGLT2i and finerenone has a synergic effect on survival, the risk of hyperkaliemia was significantly reduced when SGLT2i was added to the MRA (14.3% vs. 10.3% for no-SGLT2i and SGLT2i users, respectively). Notably, the risk of sever hyperkalemia (>6.0 mmol/L) was also reduced to one quarter of non-SGLT2i users (3.4% to 0.9% for no-SGLT2i and SGLT2i users, respectively). In the Dapagliflozin and Prevention of Adverse Outcomes in Chronic Kidney Disease (DAPA-CKD) trial, 5.3% of 4304 patients were receiving steroidal MRA spironolactone or eplerenone at baseline. The benefit of dapagliflozin on primary and prespecified kidney-specific secondary endpoints was similar in MRA users and non-users, as evidenced by the incidence of hyperkalemia (≥6.0 mmol/L) events [106]. These data are coherent with the secondary analysis of the Dapagliflozin And Prevention of Adverse-outcomes in Heart Failure trial (DAPA-HF) trial in which 71% of patients received dapagliflozin and a MRA and moderate-to-severe hyperkalemia events (>6.0 mmol/L) were reduced by 50%. Similar results were obtained in the Empagliflozin Outcome Trial in Patients with Chronic Heart Failure with Preserved Ejection Fraction (EMPEROR-preserved) trial, in which 37.5% of patients received empagliflozin and a MRA [107], and, although to a lesser extent, in the Empagliflozin Outcome Trial in Patients With Chronic Heart Failure With Reduced Ejection Fraction (EMPEROR-reduced) trial, in which 71% of patients received empagliflozin and an MRA [108]. Importantly, the FIDELITY analysis also revealed that patients who were taking both finerenone and an SGLT2 inhibitor (SGLT2i) at the beginning of the study did not exhibit an elevated risk of acute kidney injury or deterioration of renal function. In fact, there was a numerical reduction in these adverse events [105].

The effects of combination of finerenone and SGLT2i is currently under investigation in the Combination effect of finerenone and empagliflozin in participants with CKD and type 2 diabetes using a UACR Endpoint (CONFIDENCE) trial (trial NCT identifier: NCT05254002, https://clinicaltrials.gov/ct2/show/NCT05254002 accessed on 28 April 2023). The study recruited individuals with type 2 diabetes (T2D), stage 2–3 chronic kidney disease (CKD), and a urinary albumin-to-creatinine ratio (UACR) of ≥300–<5000 mg/g. These participants were receiving treatment with the maximum tolerated dose of an ACE inhibitor (ACEi) or an angiotensin receptor blocker (ARB). Importantly, they had not previously received treatment with either finerenone or empagliflozin, ensuring they were treatment-naive for these specific medications. The study investigated whether dual therapy is superior to finerenone alone and/or empagliflozin alone in reducing UACR after 6 months [109]. Secondary endpoints will also assess the additional efficacy and safety of the combination treatment and results are expected for the first quarter of 2025.

In conclusion, although there are no experimental data from specific RCTs and previous RCTs evaluating combination therapies acting on the blockade of RAS pathway resulted in safety concerns [82,110,111], the preclinical data and results of several sub-analyses suggest that the combination of finerenone with SGLT2i is a promising therapeutical approach able to provide an additional renoprotection effect with no increase of adverse events. 

Finally, it could be interesting to test potential synergic interactions between finerenone, SGLT2-inhibitors, and aldosterone synthase inhibitors that have been demonstrated to minimize aldosterone effects, especially in hypertensive patients, without hyperkalemia as a side effect [112]. In particular, next-generation aldosterone synthase inhibitors are more selective for aldosterone synthase. The most selective inhibitor in preclinical studies comes from Boehringer Ingelheim (BI 689648 and BI 689794), which had more than 300-fold selectivity compared to older aldosterone synthase inhibitors K [113]. This treatment option needs to be developed for clinical studies in DKD and it can be speculated that a combination of these drugs with SGLT2-inhibitors and finerenone could work on inflammatory and fibrotic complications of diabetic cardionephropathy.

## Data Availability

Not applicable.
